# Clinicians’ Real World Perceptions of Pre-Nephrectomy Diagnostic Biopsy Performance as a Driver of Reduction in Unnecessary Surgeries in Renal Tumors

**DOI:** 10.15586/jkcvhl.2015.20

**Published:** 2015-01-18

**Authors:** Kristin Fahy, Lauren Augustine, Mats O. Sanden, E. Robert Wassman

**Affiliations:** 1 Impetus Health LLC, Utica, New York, USA; 2 Kyruus, Boston, MA, USA; 3 Rosetta Genomics, Philadelphia, PA 19104 USA; 4 Rosetta Genomics, Princeton, NJ 08945 USA.

## Abstract

Operative removal of oncocytomas is generally unnecessary, but not infrequent in the context of renal masses. The infrequent use of pre-nephrectomy biopsies is a function of historical limitations of histopathological differential diagnosis in this setting. Assessment of clinicians’ receptiveness to a novel molecular diagnostic approach to this challenge was undertaken by means of a survey vehicle administered to 102 practicing urologists and pathologists who met inclusion criteria related to their actual clinical activity. Survey results supported the previously reported observations on misdiagnosis with urologists’ reported rates of 25% inconclusive results, and an additional 17% disagree with the final surgical diagnosis. The self-reported rate of 9% for pre-operative biopsies was comparable to prior reports, but 39% of urologists who are not currently performing pre-operative biopsies expressed interest in introducing them into their practice for this purpose with an improved diagnostic. Almost all urologists (94%) felt it important not to resect benign oncocytomas and 62% indicated they would use a test which improved the ability to sub-type renal tumors pre-operatively. The level of performance benchmark of the unidentified prototypic microRNA-based diagnostic as reported previously in the literature was deemed sufficient to change care in these cases by 73%. Overall they predicted a 38% rate of biopsies and resulting increases in decisions to forgo nephrectomy or to perform only partial nephrectomy. Pathologists also expressed support for the use of this technology in the context of inadequate specimens and for improved sub-typing of these tumors in inconclusive cases.

## Introduction

Renal cell carcinomas (RCC) are a family of carcinomas that arise from the epithelium of the renal tubules, and account for more than 3% of adult malignancies. The estimated number of new U.S. cases has reached over 65,000 in recent years, and is the cause of death for over 13,000 annually (1–5). The incidence of RCC has increased significantly in recent years (2), likely due to a higher rate of incidental findings on abdominal imaging studies done for nonspecific abdominal complaints. RCC is characterized by a lack of early warning signs, diverse clinical manifestations, and tumor resistance to radiation and traditional chemotherapy. The longstanding mainstay of treatment is radical nephrectomy, however subtotal nephrectomy is being increasingly employed in part due to the identification of smaller tumors earlier in their course by imaging (5,6).

Renal Oncocytoma (RO) is the most common benign tumor of the kidney, and is often misdiagnosed as RCC because of similar radiological and histological appearance. In particular, the Chromophobe RCC (chRCC) subtype may be challenging to discriminate from oncocytomas histopathologically. Three recent review articles (7–9) conclude that to date, histochemical, IHC, and genetic features have not proven to be reliable and specific enough markers for differential diagnosis of chRCC versus RO.

ChRCC has many biological similarities to oncocytomas and both seem to have a different origin than papillary and clear cell (aka conventional) RCCs. While chromophobe cancers may have a somewhat more favorable prognosis than the other RCC subtypes, they are nonetheless malignant tumors with the potential for metastasis and death. Oncocytomas have now been consistently classified as benign, and appropriate for conservative management, with monitoring and expectant treatment rather than total nephrectomy. Only a single case report of metastases from ROs remains confirmed in the literature, with the absence of other cases strongly supporting this conservative approach (7).

Imaging characteristics, including tumor size, alone has historically been a major factor in pre-operative diagnostic assessment to predict likelihood that a renal mass is malignant, but no imaging approach has been sufficient to adequately and fully differentiate these masses. In fact, increased detection of smaller tumors incidentally is creating confusion and adding to the difficulty in presenting options to patients regarding intervention. In addition, some small renal masses found to be chRCCs today may, in some situations, be suitable for active surveillance similar to ROs rather than immediate resection or ablation (8).

A recent study (6) showed that approximately 20% of nephrectomies performed for presumed RCC were in fact ROs on post-operative pathological evaluation, which remains consistent with past series cited. This is attributed at least in part to the fact that less than 10% of patients undergo a pre-operative diagnostic biopsy prior to having a nephrectomy for a presumed renal cancer (6). These low rates of pre-operative biopsy are largely attributed to less than desirable diagnostic accuracy by histology and immunohistochemistry in this setting (9, 10).

MicroRNAs are small non-coding RNA molecules that play an important role in the regulation of gene expression (11–13), which have been increasingly established as strong molecular biomarkers for clinical diagnostics (14–19). The tissue-specificity of microRNA expression opens a window to new types of molecular diagnostic assays, and its utility in kidney cancer classification and diagnosis has recently been described (20–22). A clinically validated microRNA-based diagnostic is now commercially available which discriminates accurately between the four most common kidney tumors, notably differentiating benign oncocytomas from chromophobe, papillary, and clear cell RCCs (22).

In order to understand the perceived unmet need, clinical utility, and projected use of an improved diagnostic for pre-operative decision making with kidney tumors, an independent third-party survey was conducted with practicing urologists and pathologists. The study’s intent was to explore the factors that influence testing for histological subtype in kidney cancer, the perceived accuracy of testing, and its impact on the decision to perform a resection. Premised upon the reported performance characteristics of a novel clinical microRNA based assay for subtyping of kidney tumors (**Table 1**), and urologists’ and pathologists’ current clinical practices, we explored their perceptions on integrating such a diagnostic tool into clinical practice, and identifying what significance such implementation may have on their decision to perform surgery and implications this may have on healthcare system costs.

**Table 1.** Projected performance characteristics of diagnostic.

Class	%cases	Sensitivity	Specificity	PPV	NPV
Clear (malignant)	73.0	98.15	99.20	99.8	95.2
Papillary (malignant)	15.7	98.04	99.25	91.9	99.6
Chromophobe (malignant)	5.2	92.86	96.50	83.3	99.6
Oncocytoma (benign)	6.1	86.50	98.00	93.4	99.1

## Methods

MedPanel, LLC (Cambridge, MA, USA) is a research organization with an extensive global community of healthcare professionals across multiple clinical disciplines within both the community and academic medical center settings. MedPanel was commissioned to develop a survey to understand the perceived needs for better diagnosis in renal tumors and the potential impact of a new biomarker test with specified performance characteristics. **(Supplementary files)**. It was a double-blinded study whereby the participating physicians were blinded throughout the research as to the identity and source of this specific test, as well as the study sponsor’s identity, and the identity of the participants was confidential to the sponsor.

The test performance profiled was shown as reported in recent publications for the Rosetta Kidney Test™ (**Table 1**) based on a blinded validation of the assay in 201 independent samples (22).

Additional information was provided that overall, as to the test’s ability to discriminate “Malignant” vs “Benign”, the sensitivity for malignant was 98% with a specificity of 86.5%, and the turn-around-time for results was approximately 7–10 days **(Supplementary Files)**.

Practicing community and academic urologists and pathologists were recruited nationwide to participate in this survey. Criteria for study inclusion was that physicians be U.S. based board-certified urologists or pathologists experienced in the diagnosis and treatment of kidney cancer in clinical practice and have over 3 but less than 30 years in practice. Urologists were required to be engaged in clinical practice at least 70% of their professional time, and manage a minimum of 5 kidney cancer patients per month. Pathologists were required to be engaged in performing clinical duties at least 30% of their professional time, and examine at least 5 biopsies per day.

Participants completed a self-administered 15–20 minute duration online survey tailored to their respective clinical discipline. The survey was fielded to over 1,000 urologists and pathologists online from November 15 – 26, 2013 and had a total of 102 total respondents who met inclusion criteria of which 52 were urologists and 50 were pathologists.

Additionally, MedPanel conducted four hour long in-depth telephone interviews with US-based key opinion leading (KOL) caliber physicians or physician-scientists between October 21 – 28, 2013 to help inform the quantitative survey research and obtain further qualitative insights regarding interest in, and likely use of the test. Interviewees were required to have participated in clinical trials on emerging RCC therapies and to be capable of discussing trends in, and state of the art in kidney cancer diagnostics.

## Results

The characteristics of the physicians participating in the survey are summarized in Table 2. Urologists participating spent an average of 95% of their time in clinical practice and has been practicing on average 17 years (Range: 5–30 years). They saw a wide range of new kidney tumor cases annually (5–300), but on average, 62 new cases were presented to them annually. On a monthly basis this represented between 5–85 unique patients managed on a monthly basis (Mean=23). The pathologist respondents had an average of 13 years in practice and 84% of their time was spent performing direct clinical duties. They examined an average of 25 primary tumor biopsies daily. Both clinician groups were spread across academic and community settings, with roughly equal numbers from the four quadrants of the U.S. geographically. The community hospital setting, as opposed to academic medical centers was represented slightly more for both types of practitioner.

**Table 2.**
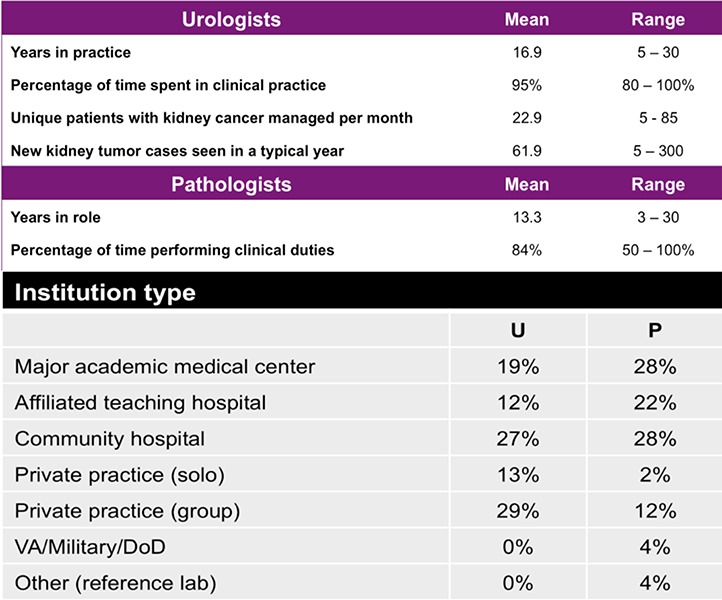
Characteristics of Survey Participants.

**Figure 1.**
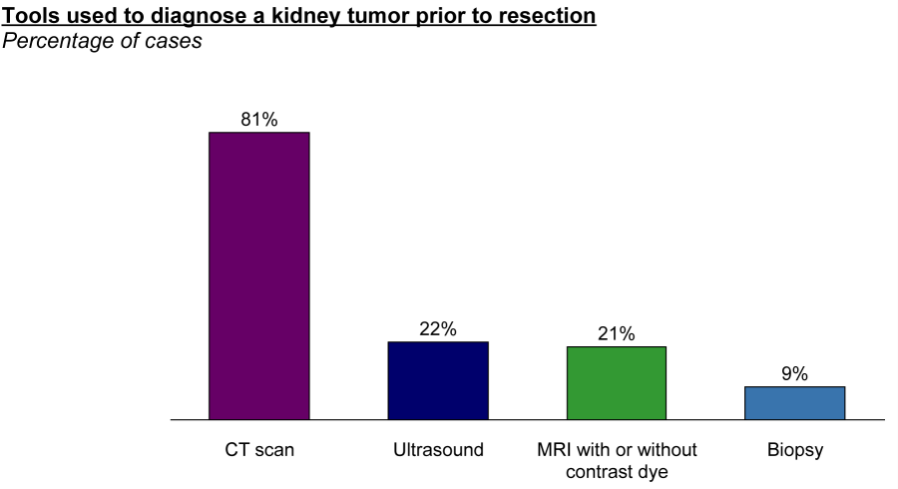
Frequency of use of pre-nephrectomy diagnostics in U.S. today.

The majority of the time, CT scans are relied upon to diagnose patients with renal masses based on size and appearance, while pre-operative biopsies are used in less than 10% of cases (**Figure 1**). Only 1 in 10 urologists routinely tests for histological subtype in pre-operative kidney tumors. Survey participants reported that they most often do not test for subtype because historically they have believed patients will require a resection regardless of subtype and/or the subtype will not change recommendation for resection.

**Figure 2.**
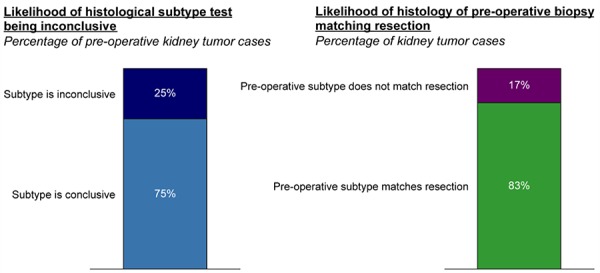
Performance of pre-operative biopsy histopathology.

**Figure 3.**
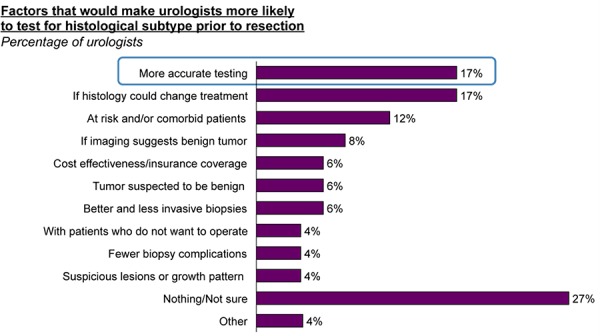
Factors that would make urologists more likely to biopsy.

Histological subtype testing on pre-operative kidney tumors is reported by respondents to be inconclusive in 25% of cases and not to match actual histology based on resection in an additional 17% of cases (**Figure 2**). Pathologists, on the other hand indicated that histology is inconclusive in only about 20% of cases. Additionally, 24% of pathologists concurred that there is a “great” need for more accurate histological testing for kidney tumors. They identified that among other factors, they would be most influenced to test pre-operatively for histological subtype prior to resection if testing was more accurate and/or it would substantively influence treatment decisions (**Figure 3**).

**Figure 4.**
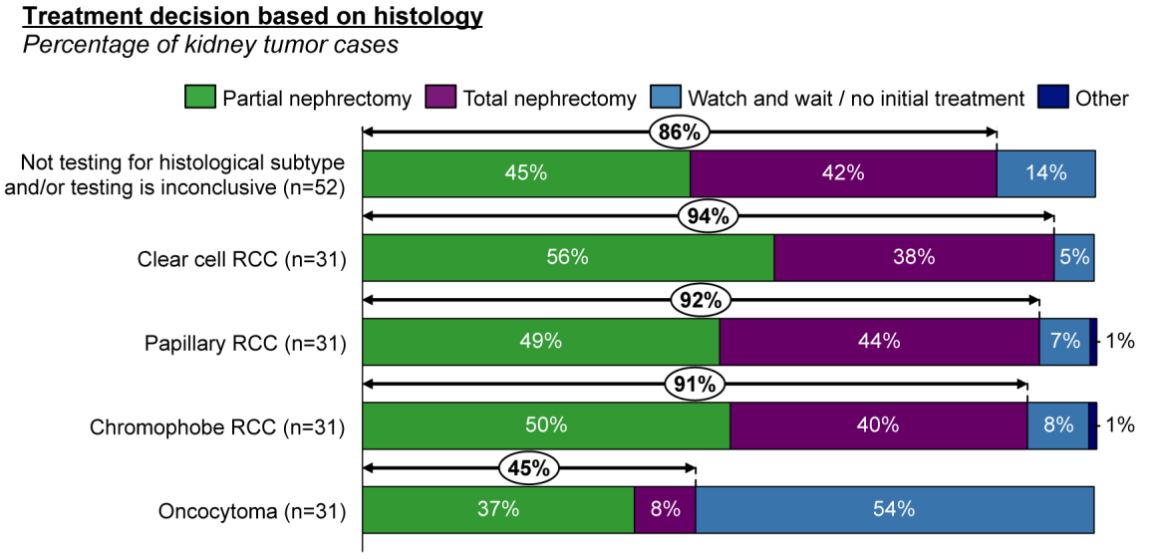
Current histological drivers of decision to resect kidney tumors.

**Figure 5.**
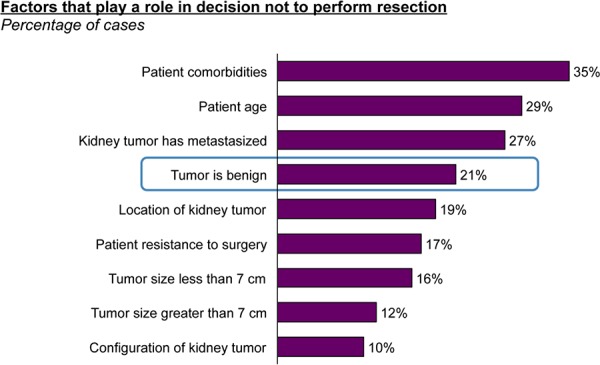
Factors influencing decisions not to resect kidney tumors.

The latter point is highlighted by the observation that when a tumor is known to be malignant, urologists would resect in over 90% of cases; and when the subtype is unknown or inconclusive, they would still resect 86% of the time. In contrast, when/if a tumor is diagnosed to be an oncocytoma, urologists report they prefer to watch and wait and would resect only 45% of such tumors (**Figure 4**). Patient wishes, and overall health and co-morbidities also factor in to decision-making regarding resection, but are largely influenced by tumor specific factors, most importantly, benign tumor type (21%) (**Figure 5**).

**Figure 6.**
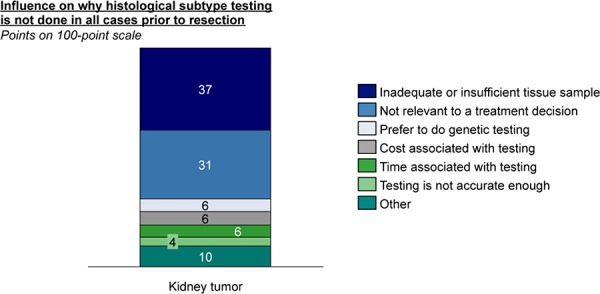
Pathologists’ rationale for not performing pre-op sub-typing.

In 8 out of 10 cases it is the pathologist who decides whether to test for subtype or not, or to do additional testing in the case of inconclusive results. Pathologists test for histological subtype in only half of pre-operative kidney tumors compared to lung tumors. They are slightly more likely to present the clinician with a “best guess” answer when it comes to kidney histology compared to lung histology, for example. Although insufficient or inadequate tissue is the major limiting factor for not performing pre-operative sub-typing in the kidney setting, (**Figure 6**) pathologists also believe subtype is not as relevant to treatment decisions. A small group of pathologists (6%) utilize genomic testing in this context to better define RCC. Overall, 24% of pathologists indicated there is a “great” need for more accurate histological testing for kidney tumors, compared to 52% who believe this for lung cancer.

**Figure 7.**
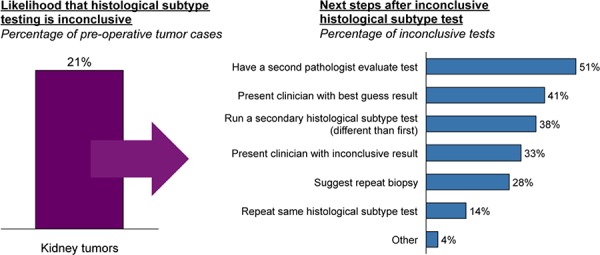
Pathologist responses to inconclusive results.

Among the 21% of cases reported to be inconclusive by pathologist respondents, just over half are referred to a second pathologist to evaluate the findings, and a second different histological test is performed in about 38% of cases (**Figure 7**). About 30% of all samples are sent for ancillary testing; however, 26% of pathologists say the need to send samples out prevents them from using a secondary test.

**Figure 8.**
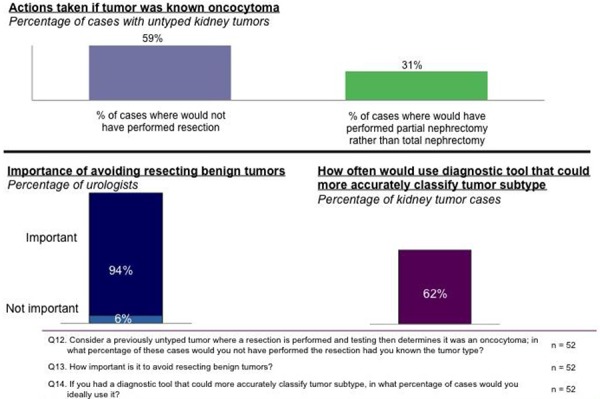
Modifications to practice with increased knowledge.

Treatment decisions today are clearly influenced by histological subtype, when known (**Figure 4**), and nearly universally, urologists (94%) believe it is important to avoid resecting a benign tumor. In untyped tumors later determined to be oncocytoma after resection, urologists would not have resected the tumor in 59% of cases if they had known the tumor was oncocytoma. Urologists stated they would use a tool that could more accurately classify tumor subtype in 62% of cases (**Figure 8**).

After reviewing the aforementioned characteristics of the Rosetta Kidney Cancer Test^TM^, 73% of urologists think it provides meaningful enough results to change treatment of oncocytoma patients. They specifically expect cases where they would not perform a resection due to an oncocytoma diagnosis to jump from 59% to 66%, and cases where they would perform a partial instead of a total nephrectomy to increase from 31% to 45%.

Overall, 64% of pathologists believed the reported microRNA-based test performance to be superior to current testing sensitivity, and 58% believe it to be superior in terms of specificity. Pathologists believe sub-type testing pre-operatively could increase from 50% to 62% of cases with availability of such a test. Somewhat more urologists (71%) believe the test’s performance is superior to current practice, and see sensitivity and specificity as most positive aspects of such a diagnostic.

Urologists and pathologists tend to see this diagnostic used as a secondary test if the first was inconclusive. Urologists were more likely to suggest the use of the test as the primary test in pre-operative tumor cases (44%), than is projected by pathologists (23%) where most still expect to use the diagnostic if initial testing is inconclusive or upon clinician request.

Urologists particularly noted the value of the diagnostic with high risk and/or comorbid patients.

**Figure 9.**
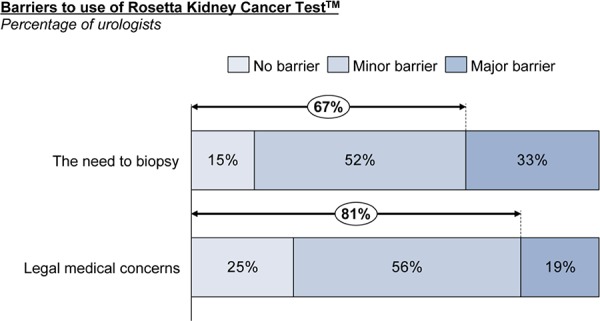
Biopsy and medical-legal concerns minimal among Urologists.

Surveyed physicians believe this test could increase testing of histological subtype as well as change treatment of oncocytoma patients. Urologists suggested that pre-operative biopsy testing could increase from 10% to 38% of kidney tumor cases. The majority of urologists do not see the need to biopsy (67%) or medical-legal concerns (81%) as major barriers to use (**Figure 9**).

**Figure 10.**
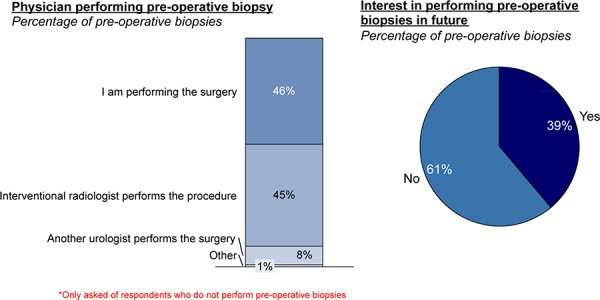
Pre-op kidney biopsy practitioners today and in the future.

When biopsies are done pre-operatively today they are roughly split between urologists (either primary or a referral) and interventional radiologists performing them. Among urologists not currently performing pre-operative biopsies, 39% expressed interest in performing them going forward prior to making a decision to resect (**Figure 10**).

**Figure 11.**
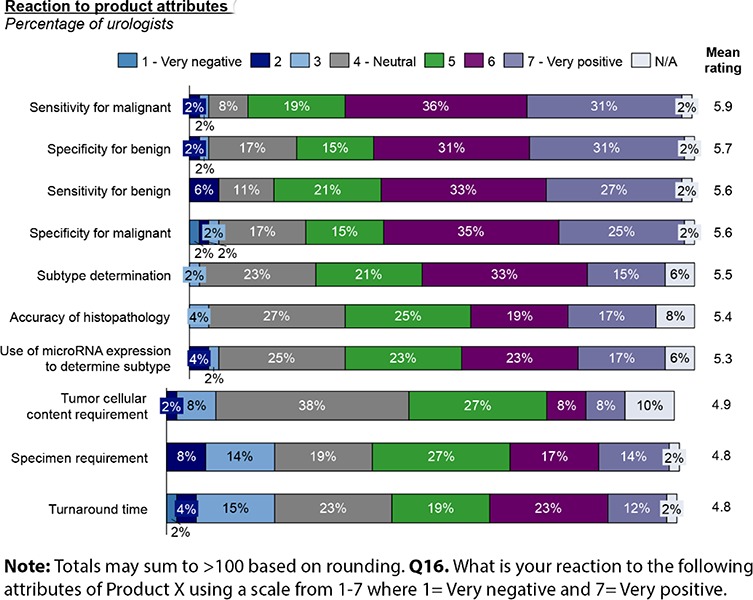
Test characteristics clinical favorability rankings.

In examining urologists’ opinions on presented performance characteristics and test logistics (**Figure 11**), turnaround time and specimen requirements regarding volume and tumor cellular content are seen as the most negative aspects of the diagnostic as presented. Despite concerns regarding specimen content, pathologists believe there would be additional tissue left for testing with this diagnostic in 45% of cases. Moreover, without a specific prompting option, 10% of pathologists say they would use the diagnostic if a priori there is inadequate tissue for histopathological and IHC studies; suggesting the test’s requirements are for less tissue in total than current tests, and may yield a diagnosis in otherwise unreportable cases.

Urologists’ perspectives on the costs and complication rates of biopsies versus total and partial nephrectomies are shown in **Table 3**, although the number of respondents in each group is sufficiently small for this to be considered only directional at most.

**Table 3.** Procedural costs and complication rates.

	**Renal Biopsy**		**Total Nephrectomy**		**Partial Nephrectomy**	
	Cost ($)	Complication Rate (%)	Cost ($)	Complication Rate (%)	Cost ($)	Complication Rate (%)
Urologist Mean	$1,222 (N=9)	5 (N=29)	$11,567 (N=9)	6 (N=38)	$7,200 (N=8)	11 (N=37)
Urologist Range	$200-$5,000	0–20	$100-$40,000	1–20	$600–20,000	1–35
AJMC 2013 (8)	ND	ND	$25,088	20%	ND	ND

Two out of four KOLs interviewed separately from the survey, believe the diagnostic would decrease the need to perform surgery; but as indicated by one KOL, this would entail a paradigm shift toward more pre-operative biopsies.

## Discussion

Despite advanced imaging techniques and the availability of pre-operative renal biopsy, most surgical series continue to report significant rates of benign lesions among renal masses resected (6,8,23,24). Preoperative biopsy of kidney tumors is not widely employed (6–9) with only about 10% of all nephrectomies preceded by a diagnostic biopsy. This problem is longstanding, and despite several academic center based studies suggesting adequate histopathological resolution is possible in this setting (25–27), diagnostic uncertainty in the differentiation of benign RO from malignant chRCC (9,10) and in additional cases even with eosinophilic clear-cell RCCs (ccRCC) remains a clear contributing factor to low rates of biopsy in routine practice. Recent reviews by Ng et al. (8) and Yusenko (9,10) both concluded that there was no reliable diagnostic marker or IHC algorithm suitable for reliable differential diagnosis of renal tumors. Ng et al. (8) concluded as had Liu and Fanning (26), that there is a group of renal tumors, notably ROs and small chRCCs, where increased confidence in diagnosis would defer or totally eliminate the need for surgical intervention.

The MedPanel survey reported here shows that practicing urologists and supporting pathologists in both academic and community settings across the United States, share the observations in the literature with their perceptions from their clinical practice. There is a similarly low rate of biopsy (9%) in our series and significant perception of inconclusive (25%) or inaccurate (17%) results from histopathological analysis of biopsies, resulting in a rate of oncocytomas in surgical series reasonably predictable to mirror that in the published literature.

There was a strongly expressed need for improved diagnostic capabilities in the preoperative setting with kidney tumors. Urologists report that if the tumor type is known to be malignant, a total or partial nephrectomy is performed over 90% of the time, and similarly if the subtype is unknown, they did the same in 86% of cases, but perform nephrectomy only 45% of the time if the tumor is an oncocytoma. Overall, the tumor being benign plays a role in making a decision not to perform a resection 21% of the time today, which underlies the expressed unmet need here.

The impact of this limitation of diagnostic capability was highlighted by a recent health economics outcome review of the management of kidney masses by Asnis-Alibozek, et al (6). This study strongly supported the adverse economic and health outcomes impact of poor pre-operative diagnosis, and confirmed the reported very low rates of biopsy. In their analysis of the IMS LifeLink database covering over 60 million commercially insured patients in the U.S., approximately 1 in 6 who underwent a nephrectomy for suspected RCC were subsequently identified as having benign disease with an economic impact of approximately $26,500 per patient overall. This is in contrast to expenditures of approximately $1,300 total pre-operatively today, most commonly for one or more CT examinations. They projected that this represents over 10,000 unnecessary surgeries in the United States alone annually.

Complications after surgery, both acute and chronic are not uncommon, although slightly less frequent in patients with benign lesions versus malignant (17.5% versus 20% respectively) and result in expenditures of over $40,000 in the year following surgery. These include such serious results as acute and end-stage renal failure requiring dialysis and kidney transplant in some, and across the board, surgical overtreatment has a profound impact on patient health–related quality of life, and outcomes. This rate was substantially lower than the complication rate of 37% in surgically treated RCC patients cited by the authors from the SEER-Medicare database, however they noted the likelihood that the SEER-Medicare data represented an older population with more co-morbidities (6).

It is noteworthy that the urologists in the MedPanel survey on average under-estimated the impact of nephrectomy in terms of costs and complication rates compared to the Asnis-Alibozek study, and by inference the SEER-Medicare population (**Table 3**). Thus the impact of the unmet need for better diagnosis identified by respondents is likely greater in these terms than the respondents appreciate (6).

Patient comorbidities often play a role in the decision to not perform a resection, as indicated by 35% of urologists surveyed. In addition to avoiding the higher costs of surgery, better pre-operative discrimination of benign oncocytomas from RCCs would avoid significant morbidity from complications and potentially result in significantly lower related health care costs.

Recent further clarity as to the appropriateness of watchful waiting in oncocytomas (28) today is likely to drive the number of patients who benefit from improved pre-operative diagnosis. Some residual hesitancy to manage ROs conservatively may remain due to the rare reported cases of metastatic oncocytomas, however these cases may in fact represent misdiagnosed chRCCs, and better diagnosis would also improve this side of the equation.

The purpose of our study was in part to assess the receptiveness of practicing clinicians to an alternative diagnostic paradigm that focuses on increased diagnostic accuracy. Avoiding unnecessary resections was clearly desirable, with 94% of urologists believing it is important to avoid resecting a benign kidney tumor. Histological subtype testing is known in only 1 out of 10 kidney tumors pre-operatively and when the subtype is unknown or inconclusive, urologists resect 86% of the time. In untyped tumors determined to be oncocytoma after resection, urologists would not have resected the tumor in 59% of cases if they had known the tumor was an oncocytoma.

Ability to inform treatment decisions would drive use of more accurate diagnostic tests. Since the impact of diagnosis of benign oncocytomas clearly impacts treatment decisions, access to a more accurate test for histological subtype would increase the use of pre-nephrectomy biopsy of kidney tumors. Urologists and pathologists concurred that the published performance characteristics of microRNA-based profiling of kidney tumors is superior to existing technology and meets their criteria to increase testing of histological subtype preoperatively with resultant significant change in how they (73% of urologists surveyed) would treat oncocytoma patients. With the availability of this test, urologists would be almost four times more likely to check for subtype in pre-operative kidney tumors, and anticipate testing would increase from 10% to 38% of cases. 44% of urologists would use the Rosetta Kidney Cancer Test^TM^ as a primary test for histological subtype, 56% would use it if a first test was inconclusive. With knowledge that a tumor is in fact an oncocytoma, cases where a resection would not be performed would increase from 59% to 66% and partial rather than total nephrectomies would go from 31% to 45%.

In summary, urologists surveyed indicated that there is 1) a need for improved pre-operative diagnosis; 2) the reported test meets their expectations in this regard; and 3) they would act upon the results of this test to decrease the number of unnecessary surgeries. The literature supports that implementation of these three facets would result in lowered morbidity and overall healthcare expenditures related to kidney tumors. Better diagnostic certainty is essential to permit medical and surgical practices to evolve and improve. It is essential to integrate new tests, which enable reductions in unnecessary surgeries based on past standards of diagnostic certainty.
